# Differentiation of the brain vasculature: the answer came blowing by the Wnt

**DOI:** 10.1186/2040-2384-2-1

**Published:** 2010-01-14

**Authors:** Stefan Liebner, Karl H Plate

**Affiliations:** 1 Blood-Brain Barrier Signaling Group, Institute of Neurology (Edinger-Institute, Frankfurt University Medical School, Heinrich-Hofmann-Str. 7, 60528 Frankfurt/Main, Germany

## Abstract

Vascularization of the vertebrate brain takes place during embryonic development from a preformed perineural vascular plexus. As a consequence of the intimate contact with neuroectodermal cells the vessels, which are entering the brain exclusively via sprouting angiogenesis, acquire and maintain unique barrier properties known as the blood-brain barrier (BBB). The endothelial BBB depends upon the close association of endothelial cells with pericytes, astrocytes, neurons and microglia, which are summarized in the term neuro-vascular unit. Although it is known since decades that the CNS tissue provides the cues for BBB induction and differentiation in endothelial cells, the molecular mechanism remained obscure.

Only recently, the canonical Wnt/β-catenin pathway and the Wnt7a/7b growth factors have been implicated in brain angiogenesis on the one hand and in BBB induction on the other. This breakthrough in understanding the differentiation of the brain vasculature prompted us to review these findings embedded in the emerging concepts of Wnt signaling in the vasculature. In particular, interactions with other pathways that are crucial for vascular development such as VEGF, Notch, angiopoietins and *Sonic hedgehog *are discussed. Finally, we considered the potential role of the Wnt pathway in vascular brain pathologies in which BBB function is hampered, as for example in glioma, stroke and Alzheimer's disease.

## Introduction

In vertebrate ontogenesis, blood vessels arise through differentiation of mesodermal hemangioblasts into endothelial cells (ECs), which assemble into so-called blood islands of the extraembryonic yolk sac and into the aortic primordia of the embryo proper [1]. The first ECs form a primitive vascular network in a process defined as vasculogenesis with subsequent growth and remodeling of preformed vessels known as angiogenesis [2]. In fact, most organs are vascularized by initial vasculogenesis subsequently followed by angiogenic sprouting.

The central nervous system (CNS) however, is exclusively vascularized by angiogenesis starting at embryonic day 9 in rodents from the perineural vascular plexus (PVP), covering the entire surface of the neural tube [2]. In the adult, vessels will supply the brain with oxygen and glucose and they assure that metabolic end products are removed to maintain tissue homeostasis. This is of upmost importance as the function of neurons and glia depends on ion-based concentration gradients established in brain fluids, i.e. cerebrospinal fluid (CSF), providing a unique milieu for electrical nerve cell communication. Consequently, the brain is protected from the free diffusion between blood plasma and the interstitium by a vascular blood-brain barrier (BBB) in order to maintain CSF homeostasis.

**Figure 1:**
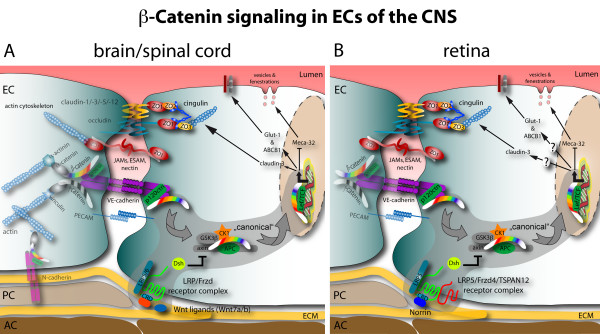
**Schematic junctional organization and β-catenin signaling in CNS ECs: comparison of brain and retina**. **A **In the brain and spinal cord mainly Wnt7a and Wnt7b growth factors act on an uncharacterized Fzd-LRP receptor complex to elicit β-catenin target gene transcription. The positively regulated target genes identified so far are the TJ protein claudin-3, Glut-1 and ABCB1/MDR1. Conversely, the expression of Meca-32/Plvap becomes suppressed by β-catenin transcription via an unknown mechanism. **B **in the retina Norrin is the predominant ligand activating β-catenin signaling downstream of the Fzd4-LRP5-TSPAN12 receptor complex. So far only the repression of Meca-32/Plvap was identified as target of this signaling. To date the effect of Norrin signaling on claudin-3, Glut-1 or other barrier-related genes is not know. See the grey supported area and arrows for the "canonical" Wnt pathway.

The mature BBB consists of a complex cellular system in which capillaries are regularly covered by a high number of pericytes embedded in a common basal membrane and by astrocytic endfeet. The circumference of brain capillaries is lined by a single endothelial cell connected with itself and neighboring endothelial cells by complex tight junctions (TJs), intermingled with non-occluding adherens junctions (AJs) [3]. AJs are formed by the endothelial specific integral membrane protein VE-cadherin, which is linked to the cytoskeleton via catenins. Endothelial expression and localization of β-catenin, γ-catenin and p120^ctn ^have been described to be crucial for the functional state of AJs including those of brain ECs (for review see [4]) (Figure 1). TJs in the CNS of mice and men are formed by occludin, as well as by members of the claudin family of transmembrane proteins [5]. In particular the endothelial specific claudin-5, which is also present in non-brain ECs, and claudin-3 were shown to localize to endothelial TJs in the CNS [6]. Moreover, claudin-1 and claudin-12 are described in brain ECs but their role is still unclear [6,7] (Figure 1). The complex TJs between brain ECs establish a high electrical resistance across the endothelial barrier (about 2000Ω × cm^2^) that is the hallmark of the BBB [8].

As ECs start to form an elaborate network of TJs during BBB differentiation, they start also to express selective transporters, such as glucose transporter type1 (Glut-1), members of the ATP binding cassette (ABC) transporter family (ABCB1/P-glycoprotein/MDR1 and ABCG2), and enzymes which together establish the complex phenotype of the BBB (for review see [9]).

In contrast to the genes, which become upregulated in brain endothelia during BBB maturation, the panendothelial cell antigen MECA-32 (also known as plasmalemma vesicle associated protein 1, Plvap1) becomes specifically down regulated [10]. As a consequence, the MECA-32 antigen is absent on the mature cerebral endothelium, whereas it remains present on vessels outside the CNS and on capillaries within the circumventricular organs (CVOs) (Figure 1).

Driven by the question what makes endothelial cells grow into the neuroectoderm, in the 1980s Werner Risau^✝ ^hypothesized that soluble factors produced by the brain and recognized by specific receptors on ECs of the PVP are responsible for brain angiogenesis. Although the initial candidates aFGF/bFGF were potent inducers of EC proliferation *in vitro *and of angiogenesis *in vivo *[11-13], their expression did not match the spatio-temporal pattern of brain angiogenesis [14-16]. When the vascular endothelial growth factor (VEGF) was identified in the brain ventricular layer, it became the most promising candidate factor specifically stimulating endothelial proliferation and sprouting via its high affinity receptors VEGF receptor 1 (VEGFR1, flt-1) and VEGF receptor 2 (VEGFR2, flk-1/KDR) (summarized in [17]). Although VEGF seems to provide the most important angiogenic stimulus also in the brain, other growth factor-receptor systems have been implicated in vascular development in the CNS. In particular, angiopoietin-1 and -2 (Ang-1, Ang-2) and their common receptor Tie2 have been shown to be involved in vascular sprouting and remodeling, namely in the adhesion to the ECM and in the recruitment of perivascular cells [18]. Furthermore, the platelet derived growth factor B (PDGF-BB) has been shown to be important for pericyte recruitment in general and interestingly, mice deficient for PDGF-BB show a complete lack of pericyte investment of brain vessels [19].

One major drawback for understanding brain angiogenesis is that these mechanisms also apply outside the CNS. Therefore, it seems unlikely that they are specifically involved in BBB differentiation creating highly specialized ECs.

The motivation for this review is drawn from recent reports indicating that in particular the Wnt ligands Wnt 7a/b, signaling via the Wnt/β-catenin pathway, could act as CNS-specific morphogens regulating particularly steps of angiogenesis and vascular differentiation [20-22].

### The players in the Wnt pathway

**Figure 2:**
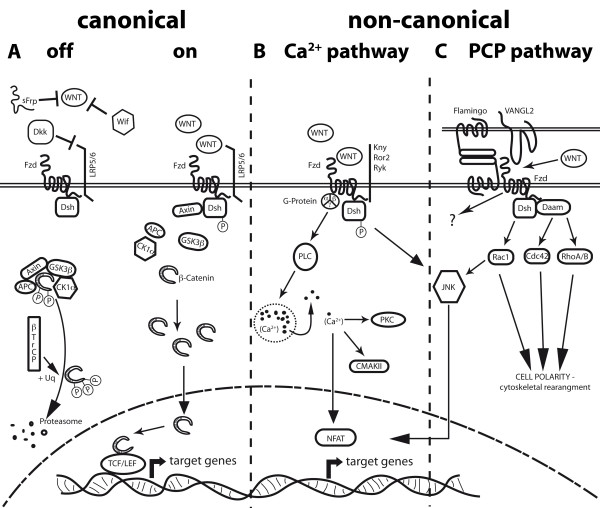
**Scheme of known Wnt pathways**. **A **Canonical Wnt/β-catenin pathway. In the "off-state" no Wnt proteins are present or are inhibited by factors like WIF, sFRPs and Dkk. Cytosolic β-catenin is targeted to proteolytic degradation through phosphorylation by the APC-Axin-GSK3β-CK1á complex and ubiquitination by the βTrCP-dependent E3 ubiquitin ligase. In the "on-state" stimulation of Fzd receptors and their co-receptors Lrp5/6 by Wnt ligands, leads to recruitment of Dvl and Axin to Fzd, thereby inhibiting the degradation complex. Consequently, β-catenin accumulates in the cytoplasm and enters the nucleus, activating target gene transcription through association with Lef1/TCF. **B **Non-canonical Wnt/Ca^2+ ^pathway. Interaction of non-canonical Wnt ligands with Fzd receptors can lead to G-Protein mediated phosphorylation of Dsh, thereby activating PLC and increasing intracellular calcium levels. These will activate CAMKII and PKC, as well as the transcription factor NFAT. Additionally, Fzd receptors in association with Kny, Ror2 or Ryk receptors can activate JNK, promoting target gene expression through AP-1. **C **Non-canonical Wnt/PCP pathway. This pathway is characterized by an asymmetric distribution of Fzds, the cadherin Flamingo and VANGL2, resulting in cell polarization. Wnt signaling promoted by either Wnt or an interaction of Fzd with VANGL2 activates RhoA/B, Cdc42 or Rac1. Dsh activates Rac1, while RhoA/B and Cdc42 need the participation of Daam1 downstream of Dsh. Rac1 can also activate JNK, resulting in the NFAT pathway. All three down stream pathways are key players in cytoskeletal rearrangement and cellular polarity. The signaling of the Flamingo/Fzd interaction is still obscure.

Wnt signaling regulates various biological processes on a cellular level such as proliferation, apoptosis, polarity and differentiation as well as pluripotency of stem cells. These functions have consequences on a tissue or organ level, influencing axis determination, segmentation as well as branching of tubular structures [23]. Wnts are a family of 19 secreted glycoproteins that primarily signal through seven-pass transmembrane receptors of the frizzled family (Fzd) [23]. Wnt proteins undergo extensive post-translational modification, such as glycosylation and palmitoylation, which influence solubility and diffusability of the proteins, and their accumulation in the extracellular matrix [24,25]. The term "Wnt signaling" comprises at least three related pathways (see Figure 2), of which the best-characterized one is the "canonical" or β-catenin (*armadillo *in *Drosophila sp*.) mediated pathway, as opposed to two "non-canonical" or β-catenin-independent pathways (see below). Canonical Wnt signaling involves binding of Wnt growth factors to the cystein-rich domain (CRD) of Fzd, which forms a receptor complex with the co-receptor LDL-receptor related protein 5/6 (LRP-5/-6, *arrow *in *Drosophila sp*.). In the absence of Wnt ligands the degradation complex, formed by Axin, glycogen syntase kinase 3β (GSK3β, *zeste-white *or *shaggy *in Drosophila), casein kinase 1α (CK1α) and adenomatous poliposis coli (APC), accomplishes the N-terminal phosphorylation of β-catenin at Ser45 (by CK1-α) and subsequently, at Thr41, Ser37, and Ser33 (by GSK-3β) [26]. Phosphorylated β-catenin becomes ubiquitinated by β TrCP (*Slimb *in *Drosophila sp*.) and degraded in the proteasome [27]. Wnt ligand binding leads by incompletely understood mechanisms to the activation of the cytoplasmic protein disheveld (Dvl), and subsequently to the recruitment of Axin to the LRP co-receptor, which results in the decay of the degradation complex, hence to the stabilization of β-catenin in the cytoplasm. Upon translocation to the nucleus, β-catenin displaces the transcriptional repressor groucho from the interaction with members of the lymphoid enhancer factor (Lef)/T-cell factor (TCF) transcriptional activators to start target gene transcription (Figure 2A) [28,29].

The non-canonical pathways, which are defined by the independence of β-catenin as a central signaling molecule, are an assembly of by now loosely separable pathways. Wnt5a, Wnt4 and Wnt11 are considered to be prototype non-canonical Wnts, which do not induce axis duplication in Xenopus. Instead, non-canonical Wnts may induce Ca^2+ ^release and activation of the protein kinase C (PKC) or Ca^2+^/calmodulin-dependent protein kinase (CamkII), both summarized as the Ca^2+^-pathway (Figure 2B). Alternatively, non-canonical Wnts can activate Rac, Cdc42, RhoA/B and downstream c-Jun N-terminal kinase (JNK) or RAB4, known as the planar cell polarity pathway (PCP). The latter has been identified in Drosophila, but is also present in vertebrate organisms and plays a crucial role in cellular convergence and extension movements during organogenesis [30]. PCP can also be activated/modulated by non-Wnt related, membrane bound Fzd ligands such as *Flamingo*, a member of the adhesion-G-protein coupled receptor (GPCR) family (mammalian orthologs CELSR1, CELSR2, CELSR3), and strabismus/Van Gogh (for review see (Figure 2C) [31]).

Moreover, secreted antagonists, including Dickkopf homologs (Dkk1-4), secreted frizzled-related protein families (sFRPs, 1-5), and atypical Wnt receptors, such as the receptor-like tyrosine kinase (Ryk) and receptor tyrosine kinase-like orphan receptors (Ror), can regulate the Wnt signaling output (see Figure 2) [32]. Furthermore, Fzd ligands, which are structurally unrelated to Wnts, such as the Norrie disease pseudoglioma protein (Nrp, Norrin), have been described to activate β-catenin transcriptional activity particularly in the vascular system and are discussed in detail in the following chapters [33].

### Wnt pathway in vascular beds of the central nervous system

So far the Wnt/β-catenin pathway has been reported to be involved in various aspects of EC biology and in angiogenesis, such as proliferation and vessel assembly. The reader is referred to the following review articles comprehensively summarizing the general role of the Wnt pathway during developmental vasculo- and angiogenesis (for review see [34-36]).

#### Vessels in the brain and spinal cord

Recently, it could be demonstrated that the combined inactivation of Wnt7a and Wnt7b leads to severely defective angiogenesis in the central nervous system (CNS) [20,22]. Wnt7a and b are expressed with overlapping domains in the presumptive forebrain and ventral neural tube. EC-specific deletion of β-catenin phenocopies the angiogenic defects in the CNS, suggesting that Wnt7a and 7b promote CNS vascularization by activating canonical Wnt signaling [20,22]. The endothelial receptors involved in CNS-specific vascular Wnt signaling remain to be identified. Notably, EC-specific deletion of β-catenin also causes angiogenic defects in the non-CNS vasculature, including vascular patterning defects in the embryo proper and placenta [37]. Additionally, β-catenin KO mice lack regular heart valve development due to abrogated endocardial Wnt signaling [38], leading to earlier embryonic lethality compared to the Wnt7a/b double KO [20,22]. As a consequence, canonical Wnt/β-catenin signaling may be involved in regulating vascular morphogenesis throughout the organism, but selective Wnt ligands and/or Fzd receptors may provide organ-specific angiogenic functions.

It is obvious that, when brain angiogenesis starts, endothelial cells get in contact with various neuroectodermal cells like neuroblasts and various glial cells and/or their precursors [39]. That endothelial cells do not show a predetermination to the BBB phenotype was elegantly shown by chick-quail xenografts in which vessels of the coelimic cavity of the embryonic chick acquired BBB characteristics when growing into the developing transplanted quail brain [40].

Although numerous aspects of BBB development in brain capillary ECs have been monitored and described, the crucial question concerning the mechanism behind the induction of this differentiation process remained to be elucidated.

In this regard, it was of major interest whether Wnts, in addition to their function in brain angiogenesis, might participate also in the differentiation of the BBB phenotype in brain ECs. Indeed, Wnt7a and Wnt7b were also shown to induce expression of the glucose transporter 1 (Glut1), an early marker of endothelial BBB specialization (see Figure 1A) [20,22].

Moreover, we demonstrated in a collaborative effort that *β*-catenin-TCF mediated Wnt signaling in ECs is necessary and sufficient to induce the mature molecular and structural properties of BBB-type endothelial TJs and barrier maturation *in vitro *and *in vivo *[21]. Activation of β-catenin transcriptional activity by Wnt3a-conditioned media or dominant active β-catenin selectively induces claudin-3 and represses Meca-32 expression, a marker for immature brain vessels, in mouse brain ECs (MBEs) (see Figure 2) [21]. In contrast to claudin-5, claudin-3 is predominantly present in brain ECs and is specifically upregulated during BBB establishment and maintenance although the specific function of claudin-3 for BBB TJs remains elusive [6]. Interestingly, ectopic Wnt7a expression by Nestin-Cre-mediated recombination in scattered cells outside the CNS was sufficient to induce Glut-1 expression in non-brain ECs [22]. Similarly, viral infection of a non-brain endothelioma cell line with dominant-active β-catenin, was sufficient to upregulate claudin-3 and to downregulate Meca-32 [21]. These findings support the results of chick-quail chimera experiments, suggesting that the specificity of BBB induction *in vivo *is achieved by the brain milieu, which we now know is at least partially defined by the selective availability of Wnt ligands, rather than by a selective receptor endowment of ECs. However, further studies are needed to clarify this issue in detail.

Furthermore, the contribution of other Wnts than Wnt7a/b to endothelial barrier induction *in vivo *remains elusive. As Wnt7a/b are predominantly expressed in the ventricular zone of the developing forebrain and in ventral portions of the neural tube, other Wnt ligands such as Wnt1, 3, 3a and 4 may take over barrier induction in dorsal parts of the neuroectodem [20].

Most of the brain capillaries develop BBB characteristics, hence induction needs to take place in the majority of ECs with the only exception in vessels of the CVOs [41]. Leaky areas, lacking an endothelial BBB, are located at strategic positions of the ventricular system, as for example in the pituitary and the choroid plexus where active secretion and resorption takes place, respectively [41]. Whether in CVOs vessels are specifically devoid of Wnt signaling during angiogenesis of the CNS has to be clarified in future investigations.

#### Vessels in the retina

During angiogenesis of the retina as part of the CNS, microvessels form the blood-retina barrier (BRB), which is structurally and functionally similar to the BBB. In the retina, Wnt signaling has been reported to regulate specification of stalk cells in the expanding vascular network [42]. Herein the Notch-regulated ankyrin repeat protein (Nrarp), a gene activated by Delta-like 4 (Dll4)/Notch1 signaling in stalk cells, promotes canonical Wnt signaling through direct interaction with Lef1 [42]. ECs lacking Nrarp show decreased levels of Lef1 and cyclinD1, and a reduced proliferation rate, indicating a possible *in vivo *role of Wnt signaling in regulating EC proliferation [42].

Fzd4 KO mice display retarded retinal and ear vessel growth and inappropriate vessel pattering, delayed hyaloid vessel regression, dead-ended aneurysm-like capillaries, vascular leakage and abnormal *corpora lutea *angiogenesis leading to infertility [43,44]. Furthermore, the Wnt-unrelated Fzd4 ligand Norrin can activate canonical Wnt signaling and the Norrin-deficient mouse displays similar defects as the Fzd4 KO [45-47].

Also inactivation of the Lrp5 gene in mice leads to delayed hyaloid vessel regression and defective migration of retinal blood vessels [48,49]. Apparently, canonical Wnt signaling is important for vessel regression in hyaloid vasculature, but also for growth and stability in retinal vessels. The work of Lang and co-workers suggest that paracrine macrophage-derived Wnt7b induces vascular regression of hyaloid vessels in the absence of the survival stimulus Ang-1 [50,51]. Therefore, the specific outcome of Wnt signaling in different vascular beds might depend on the microenvironment and crosstalk between different signaling pathways and survival signals, coordinating EC proliferation and apoptosis, vessel migration and regression (Wnt, VEGF-A and Ang-1/-2).

Another interesting question is how barrier properties are induced in retinal blood vessels, which form the BRB. Recently, Norrin has been implicated in this function [52]. Junge and co-workers showed that Norrin signals via the receptor complex Fzd4-Lrp5, which is specifically clustered by tetraspannnin 12 (Tspan12), thereby enhancing β-catenin transcriptional activity (Figure 2). Interestingly, Norrin-mediated signaling downregulates the expression of Meca-32, which inversely correlates with barrier formation. Furthermore, Luhmann and colleagues showed that norrin-deficient mice exhibit reduced vascular density in the cerebellum, suggesting that norrin regulates vascularization also in the cerebellum [53]. It is tempting to speculate that norrin also directly regulates barrier properties in retinal and cerebellar ECs as shown for Wnts in ECs of other CNS regions [20-22].

Although by now the involvement of Wnt/β-catenin signaling in brain angiogenesis and endothelial barrier induction has been established, it remains to be clarified to which extend the plethora of BBB associated genes are directly or indirectly dependent on the Wnt pathway. In this regard Lim and co-workers described the up-regulation of P-glycoprotein (also know as MDR-1, ABCB1) upon GSK3^β ^inhibition in immortalized human ECs [54], supporting the idea that Wnt/^β^-catenin signaling regulates induction and maintenance of BBB genes also in human ECs.

#### Crosstalk with other pathways in the endothelium

It is known that Wnt signaling does not regulate cell and tissue differentiation on its own, but interacts with other major pathways, which play a crucial role herein. Although by now we are still scratching the surface of the Wnt pathway in endothelial biology, it is more than likely that extensive crosstalk to pathways such as receptor tyrosine kinases (VEGF/VEGFR, FGF/FGFR), Notch, TGFβ/BMP and *Sonic hedgehog *(Shh) will be brought to light by future work.

Below the currently known as well as some hypothetical interactions are discussed. The most prominent finding for vascular biology was that the Wnt pathway regulates VEGF in colon carcinoma [55]. This has also been confirmed in human umbilical vein ECs (HUVECs) infected with a kinase-mutant form of GSK3β, leading to increased β-catenin signaling and downstream VEGF expression [56]. VEGF acts in an autocrine manner in HUVECs, thereby activating the PI3K/Akt pathway, leading to vascular sprouting and network formation in matrigel *in vitro*.

Conversely, PI3K/Akt signaling, which is activated by receptor tyrosine kinases (RTKs), may positively regulate β-catenin transcriptional activity independent of Wnt-Fzd interaction [57].

The process of vascular sprouting is regulated by numerous signals and recent studies identified the Dll4/Notch1 signaling axis as the key endothelial mechanism controlling the appropriate numbers of sprouting tip and following stalk cells (for review see [58]). Interestingly, Nrarp, which is a direct target of the Notch pathway in stalk cells, interacts with β-catenin and stabilizes the β-catenin/Lef/TCF transcription complex, thereby rendering β-catenin transcription active [42,58]. As in stalk cells Jagged-1 is specifically expressed as opposed to Dll4 in tip cells, it is tempting to speculate that Jagged-1 could be regulated by β-catenin/TCF signaling in ECs, as it has been described in epithelial cells of the skin and colon [59,60].

Wnts, and in particular Wnt7a/b have recently been implicated in vascularization of the developing neuroectoderm [20,22]. Similarly, *Sonic hedgehog *(Shh) seems to upregulate angiogenic factors [61] and to be important for vascularization of the neural tube in an Ang-1-dependent manner [62]. The availability of Ang-1 was previously discussed to be crucial for the cellular response of hyaliod vessels to macrophage-derived Wnt7b [50,51]. Whether Wnt7a/b or any other Wnt ligand functionally interact with Shh to regulated brain vascularization is still obscure. However, ventral Shh signaling in the neural tube is important to maintain dorsal β-catenin/TCF signaling on a physiological level [63]. Whether this applies also to the angiogenic blood vessel needs to be clarified.

There is ample evidence that the NF-κB pathway and the Wnt/β-catenin pathway interact to differentially regulate inflammation, tumor formation, metastasis and resistance to chemotherapy in epithelial cells [64]. Apparently, GSK3β plays a central role in this interaction, as GSK3β is a negative regulator of β-catenin but positively regulates NF-κB by targeting IκB, the major inhibitor of NF-κB, to proteasomal degradation [65]. Furthermore, β-catenin itself can form a complex with the p50 subunit of NF-κB, thereby preventing NF-κB transcriptional activity [66]. In ECs both pathways play a major role in angiogenesis, inflammatory response and vascular homeostasis and although interaction has not been confirmed so far it is likely that Wnt/β-catenin and NF-κB also interact in the endothelium.

Recent studies link Wnt/β-catenin to hypoxia signaling by the expression of von Hippel Lindau (VHL) via the β-catenin-TCF-4 complex [67]. VHL is an ubiquitin ligase involved in degradation of hydroxylated hypoxia inducible factor-1α (HIF-1α). In turn HIF-1α can directly bind to β-catenin, competing with TCF-4 and thereby enhancing hypoxia-mediated transcription [68]. Whether the very same mechanism operates in ECs remains to be shown. Interestingly, searching for astrocytic BBB-maturation factors regulated by oxygen, Lee and colleagues identified the potential tumor suppressor *src*-suppressed C-kinase substrate (SSeCKS, spoken "essex", human ortholog is gravin), which was upregulated upon increasing oxygen tension [69]. The overexpression of SSeCKS in astrocytes markedly downregulated VEGF and upregulated Ang-1 expression *in vitro*, thereby leading to increased junction localization of ZO-1 and claudin-1 in ECs. Furthermore, SSeCKS can be induced in astrocytes by PDGF-B, which in turn is produced by endothelial cells and plays also an important role in pericyte recruitment to blood vessels [70].

It is tempting to speculate that a molecular link between Wnt and hypoxia signaling may also be relevant for the endothelial cell response in physiological and pathological sprouting angiogenesis.

### Endothelial Wnt pathway in CNS pathologies

Since Wnt signaling was identified to play a major role in vascular development and differentiation, it is of particular interest to understand its involvement in pathological angiogenesis in which hypoxia is known to be one of the major driving forces.

Tissue hypoxia takes also place in tumors and is described as a prerequisite for the angiogenic switch of various cancers, leading to the massive expression of VEGF-A by hypoxic tumor cells [71]. The most frequent and most malignant primary brain tumors of adulthood are WHO grade IV astrocytoma (glioblastoma), which are known to be highly hypoxic and extensively vascularized [72]. Recent reports suggest that β-catenin signaling in glioma cells participates in tumor cell progression and invasion [73-75]. This effect is, at least partially, mediated by the epidermal growth factor receptor (EGFR), which activates ERK leading to the dissociation of α- and β-catenin, thereby promoting β-catenin transactivation [73]. To which extend glioblastoma cells express Wnt growth factors, leading to autocrine and/or paracrine signaling to the vascular compartment, as it is known from VEGF, is poorly known.

Nevertheless, glioblastoma tumor vessels were demonstrated to regularly show nuclear β-catenin in human samples and in an N-ethyl-N-nitrosourea-induced rat glioma model [76,77]. The specific function of endothelial β-catenin signaling in glioblastoma however has not been determined so far.

After stroke, independent of its etiology from ischemic or hemorrhagic insult, BBB integrity is focally abolished and during glia scar formation, inflammation and neo-angiogenesis occurs [78]. Lithium chloride (LiCl), which is a widely used inhibitor of GSK3β, was frequently applied to treat bipolar mood disorders. More recently, LiCl was implicated in the reduction of infarct volume in a rat model for stroke [79]. After middle cerebral artery occlusion (MCAO), LiCl had a beneficial effect on the survival of neurons up to 3 h after onset of stroke, but the effect on EC has not been determined. Interestingly, Guo and co-workers have shown that LiCl upregulates VEGF-A in human brain microvascular ECs and astrocytes *in vitro *through PI3K/GSK-3β-dependent and -independent pathways, respectively [80]. This might contribute to the beneficial effect of LiCl treatment on vascular remodeling after stroke [81]. VEGF in turn is a described Wnt/β-catenin target and consequently, β-catenin signaling might have multimodal effects for the prevention of the destructive effects elicited by stroke. Not to forget the possibility that LiCl via β-catenin activation, may help to maintain and/or re-induce barrier properties in brain microvessels of the infarct area.

Finally, the familial form of Alzheimer's disease (AD), in which the presenilin-1 (*PSEN1*) gene is mutated, has been correlated with reduced levels of active β-catenin [82]. And recently, amyloid-beta peptide (*Aβ*), the major product of the presenilin/γ-secretase complex, was demonstrated to bind to the CRD of Fzd, thereby inhibiting Wnt mediated signaling [83]. This may further correlate with the known BBB defects in AD, which by now seem to be related to decreased clearance of *Aβ *by EC-specific transporters (reviewed in [41]). Herein the β-catenin target gene P-glycoprotein (MDR-1, ABCB1) appears to play the major role in ECs.

Important clinical insight into the involvement of Wnt signaling in vascular pathogenesis came from studies of the human disorders Norrie disease (ND), Coat's disease and familial exudative vitreoretinopathy (FEVR) as mentioned above [84]. FEVR has been linked to 4 different loci, EVR-1 to 4, three of which encode for Norrin, Fzd4 and Lrp5 [46].

Norrie disease is caused by a X-linked recessive mutation of Norrie disease pseudoglioma gene (NDP), coding for Norrin/Norrie disease protein (Ndp) [85,86]. The mouse Ndp KO displays a lack of deep retinal capillary networks and subsequent retinal hypoxia, which is accompanied by an increased expression of the angiogenic factors VEGF-A, β3 integrin and Tie1 [45]. Norrie patients suffer from blindness of both eyes at birth or soon after, leukocoria and cateract formation. Additionally, they develop sensorineural hearing loss, impaired motor skills and mental retardation caused by vascular defects in the inner ear and the cerebellum (reviewed in [87]).

In contrast to NDP mutation, Fzd4 mutations are autosomal dominant and those of Lrp5 are autosomal recessive, but phenotypes are largely overlapping.

With the identification of TSPAN12 as a modulator of Norrin signaling that genetically and functionally interacts with Fzd4, another gene could potentially be mutated in the FEVR family of diseases, although no mutation has been discovered in humans so far [52].

## Conclusions

The underlying mechanisms for CNS vascularization in general and for endothelial barrier differentiation in particular have been obscure for about one century, since Paul Ehrlich and Edwin Goldmann established the concept of the BBB. The recent identification of the Wnt/β-catenin pathway as a driving force for brain angiogenesis and BBB differentiation has shed first light onto this burning question. Although Wnt7a/7b were confirmed to be the major ligands activating β-catenin signaling in brain and spinal cord ECs, it remains elusive if exactly the same molecular pathway operates in the entire CNS to induce the BBB phenotype in ECs. This hypothesis is challenged in the retina, in which Norrin via Fzd4-Lrp5-TSPAN12 appears to be the predominant activator of β-catenin signaling in ECs. The finding that ECs can express the majority of Wnt pathway related genes *in vitro *and/or *in vivo *makes the interpretation of signal specificity even more complicated.

Furthermore, Wnt signals in the brain vasculature will integrate with other pathways such as Notch, FGF, TGFβ and *Sonic hedgehog *to regulate BBB formation. The challenges for the further understanding of brain angiogenesis and endothelial barrier differentiation are the identification of cell and tissue specificity of the Wnt pathway for CNS vascular physiology, as well as the definition of Wnt signaling as a *bona fide *target to modulate barrier properties under pathological conditions.

## Competing interests

The authors declare that they have no competing interests.

## Authors' contributions

SL was involved in preparation of the manuscript. All authors read and approved the final manuscript.
